# Flexible transnasal endoscopy with white light or narrow band imaging for the diagnosis of laryngeal malignancy: diagnostic value, observer variability and influence of previous laryngeal surgery

**DOI:** 10.1007/s00405-018-5256-1

**Published:** 2018-12-19

**Authors:** Nikolaos Davaris, Susanne Voigt-Zimmermann, Siegfried Kropf, Christoph Arens

**Affiliations:** 1 Department of Otorhinolaryngology, Head and Neck Surgery, Magdeburg University Hospital, Magdeburg, Germany; 2 Institute of Biometry and Medical Informatics, Otto-von-Guericke University, Magdeburg, Germany

**Keywords:** Laryngeal endoscopy, Narrow band imaging, Observer reliability, Laryngeal cancer

## Abstract

**Purpose:**

Flexible transnasal endoscopy is a common examination technique for the evaluation of laryngeal lesions, while the use of narrow band imaging (NBI) has been reported to enhance the diagnostic value of white light endoscopy (WLE). The purpose of this study is to assess observer variability and diagnostic value of both modalities and investigate the possible influence of previous laryngeal surgery on the detection rates of laryngeal malignancy.

**Methods:**

The study was based on the retrospective evaluation of 170 WLE and NBI images of laryngeal lesions by three observers in a random order. The histopathological diagnoses serve as the gold standard.

**Results:**

In identifying laryngeal malignancy, the sensitivity of NBI proved to be higher than that of WLE (93.3% vs. 77.0%). NBI was also superior to WLE in terms of accuracy (96.3% vs. 92%) and diagnostic odds ratio (501.83 vs. 120.65). Both modalities had a specificity of 97.3%. The inter-observer agreement was substantial (kappa = 0.661) for WLE and almost perfect (kappa = 0.849) for NBI. Both WLE and NBI showed a high level of intra-observer agreement. The sensitivity was significantly lower in images with history of previous laryngeal surgery compared to those without.

**Conclusions:**

Flexible transnasal endoscopy has been proved to be a valuable tool in the diagnosis of laryngeal malignancy. The use of NBI can increase the sensitivity and observer reliability in that context and can also provide a diagnostic gain in cases with previous laryngeal surgery

## Introduction

The endoscopic diagnosis of laryngeal lesions has substantially evolved in recent years due to various technological developments, such as improvements in image resolution and the introduction of optical image-enhancement modalities. The use of flexible transnasal endoscopy can provide important clinical benefits in that context as it is timesaving, well tolerated by patients, requires no general anesthesia, and allows close examination of the laryngeal mucosa and surrounding areas. A clear clinical distinction between benign and malignant lesions during examination is of crucial importance, as the latter require prompt histopathological verification and therapy.

Narrow Band Imaging (NBI) is an optical modality based on the use of narrow band optical filters (wavelengths with peaks at 415 nm and 540 nm) corresponding to the absorption peaks of haemoglobin. Consequently, it allows better visualization of mucosal and submucosal vessels and clearer demarcation of mucosal abnormalities compared to standard white light endoscopy (WLE) without the use of dyes, as in optical chromoendoscopy [1–3]. NBI can be incorporated into rigid and flexible endoscopes, with switching between WLE and NBI modes enabled by the press of a button. Since its introduction to otorhinolaryngology by Muto in [4–6], NBI has continuously provided promising results in various clinical fields, including the diagnosis of laryngeal lesions.

Although some studies support using NBI in the context of early diagnosis of laryngeal cancer, only a few have exclusively evaluated laryngeal lesions, and the published data concerning observer variability and further influencing factors, such as the presence of previous laryngeal surgery, is scarce [7, 8]. Furthermore, since most studies use different inclusion and exclusion criteria, only partial comparisons are possible [9]. Besides that, as the endoscopic assessment can vary according to clinical experience, multiple observer studies are needed to confirm the possible diagnostic advantages of NBI.

The purpose of this study is to assess the inter- and intra-observer variability and diagnostic value of WLE and NBI in the differentiation between benign and (pre)malignant laryngeal lesions in flexible transnasal endoscopic examination. A further aim is to investigate if previous laryngeal surgery can influence the diagnostic value of WLE and NBI.

## Materials and methods

This study is based on clinical, histopathological and endoscopic data routinely collected in the Department of Otorhinolaryngology, Head and Neck Surgery of Magdeburg University Hospital, a tertiary referral hospital. The study protocol met the criteria of the Declaration of Helsinki in its latest version and was reviewed and approved by the local ethics committee (Report No. 108/14). Informed written consent was obtained before inclusion.

The examined population included all patients who underwent microlaryngoscopy with biopsy of a laryngeal lesion (either glottic, subglottic or supraglottic) in a 3 years period (01/2012–12/2015). The endoscopic images in WLE and NBI were routinely documented in the preoperative workup during flexible transnasal endoscopy after applying xylometazoline spray 4% for topical anesthesia. The equipment included an Evis Exera Videosystem (Olympus Medical Systems, Hamburg, Germany) and a high-resolution videorhinolaryngoscope with a 3.6 mm diameter insertion tube. The xenon light source had an integrated NBI-filter, allowing instantaneous switching from WLE to NBI without changing the endoscope. The histopathological reports, endoscopic images and further patient data were retrospectively acquired from the electronic patient records.

**Fig. 1:**
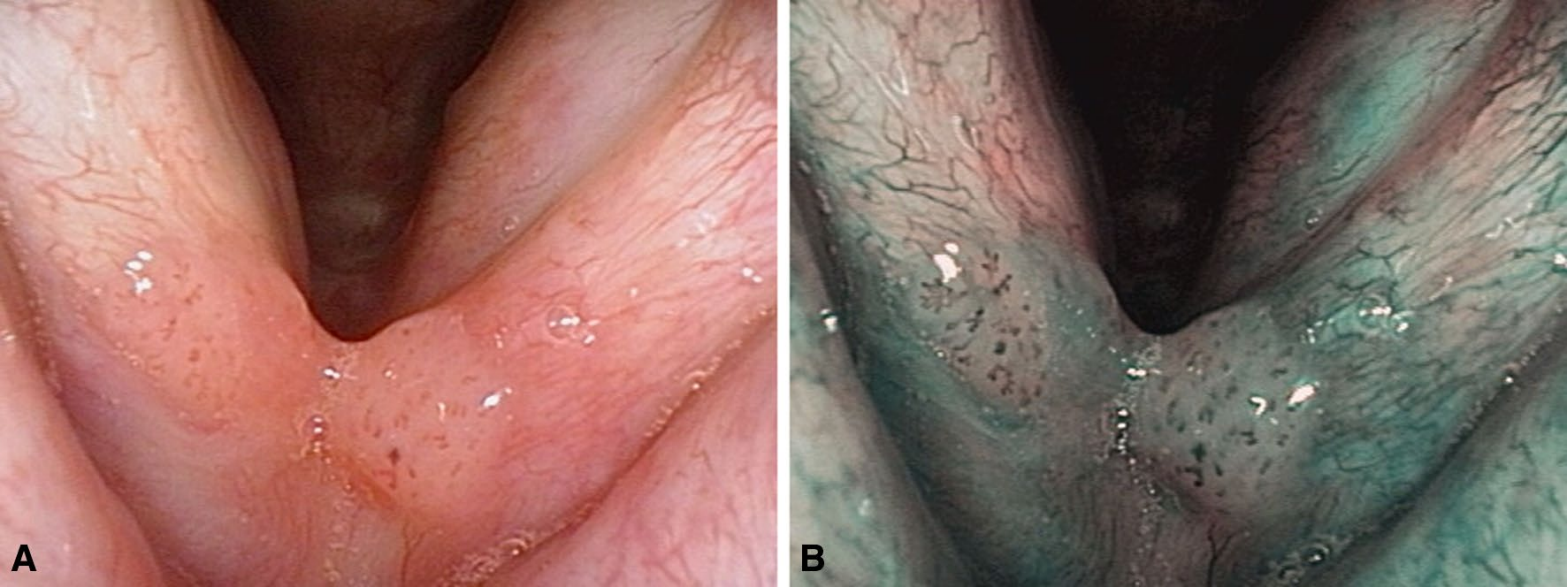
Papillomatosis of the anterior commissure **a** in white light endoscopy, **b** in narrow band imaging: homogenous multifocal volume increase with clear margins, perpendicular vascular changes inside the lesions with a central capillary loop in every morula-like bulge, several afferent hypertrophic vessels with branching

**Fig. 2:**
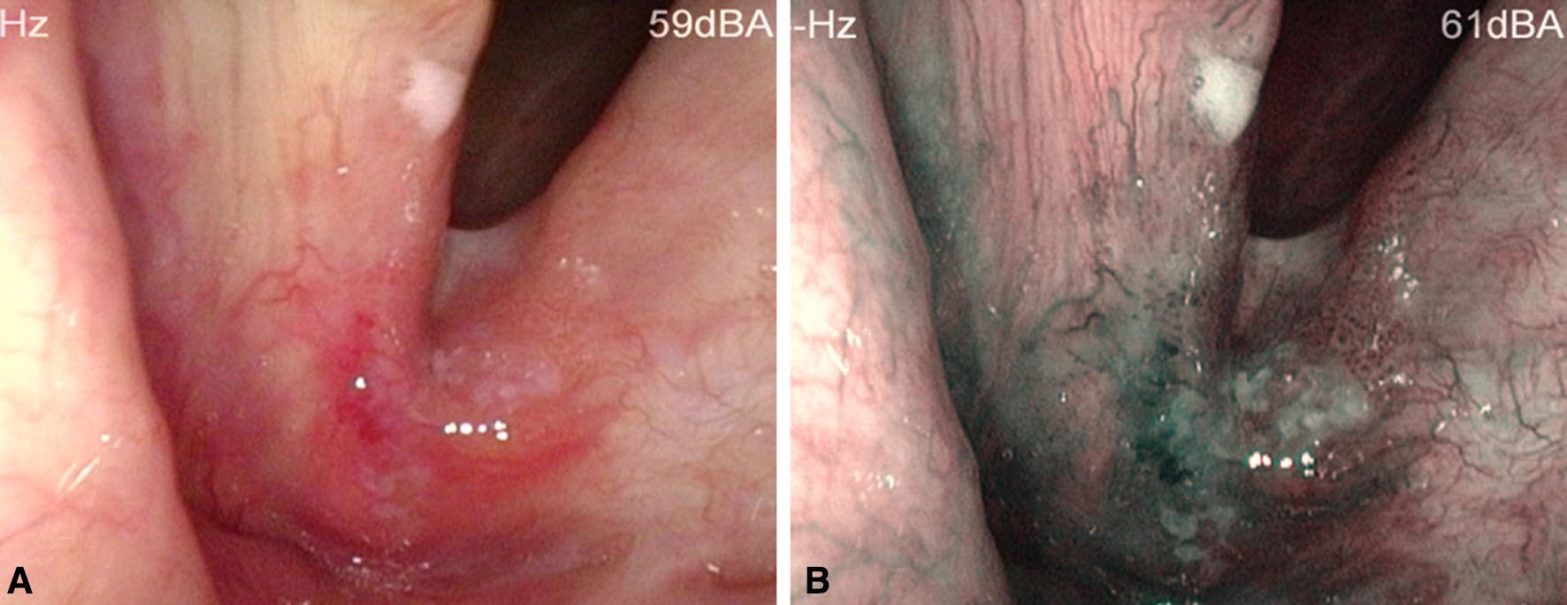
Invasive carcinoma of the anterior commissure **a** in white light endoscopy, **b** in narrow band imaging: irregular, non-homogenous leukoplakias with no clear margins, widely-spread perpendicular vascular changes with irregular distribution in and around the demarcated area, several afferent hypertrophic vessels with change of direction and branching

The inclusion criteria encompassed: availability of an image of the respective laryngeal lesion in good quality both in WLE and in NBI mode (Figs. 1, 2) and the availability of a histopathological report with a definitive diagnosis classified following the recommendations of the World Health Organization [10]. The data collected included patient demographics (age at diagnosis and sex), the localization of the laryngeal pathology, previous laryngeal surgery and the histopathological diagnosis. Incomplete patient records, insufficient image quality and/or the absence of a written consent led to exclusion from the study.

All images collected were evaluated in a random order by three observers: board-certified otorhinolaryngologists who were blinded to the histological diagnosis. The observers had 10–12 years of work experience in a tertiary referral hospital. They received training prior to image evaluation, encompassing the principles of NBI endoscopy, endoscopic appearances of healthy and pathological laryngeal tissue with NBI, and relevant image examples complete with the endoscopic criteria of malignancy, as described in the literature. The main criteria of laryngeal malignancy were: demarcated brown areas with irregular brown spots scattered in the lesion [11, 12]; exophytic or ulcerative areas and irregular brownish spots or loops depicting perpendicular vascular changes [13]; and irregular non-homogenous leukoplakias with no clear margins and/or irregular volume increase of the vocal fold (Fig. 2) [14].

The observers were required to evaluate the macroscopic characteristics of the lesion in every image and make a clinical diagnosis. Thirty-one images were selected pseudo-randomly, controlling for the distribution of malignant and benign diagnoses and presented for a second reading after an interval of 3–4 weeks to control for intraobserver agreement. All clinical diagnoses made by the observers and all histopathological diagnoses where summarized separately in two categories, malignant and non-malignant, to perform statistical analyses. For the purpose of this study, invasive carcinomas, carcinomata in situ and severe dysplasias were considered malignant, while moderate or mild dysplasias, chronic inflammation, simple hyperplasias and all histologically benign lesions were considered non-malignant.

The sensitivity, specificity and accuracy of WLE and NBI in the detection of malignant lesions were calculated for each observer and as mean values. The histopathological diagnoses served as the gold standard for all statistical analyses. For comparisons between WLE and NBI, rates of correct classification were computed for each image as an average over the three observers for each modality and then compared between the modalities with the Wilcoxon signed rank test. Confidence intervals were determined with the normal approximation for mean values. The diagnostic odds ratio (DOR) was extracted for WLE and NBI as a single indicator for each test’s performance [15] separately for each observer and also as a mean value.

The inter-observer agreement for each optical modality (WLE and NBI) was calculated using Fleiss’ kappa for use with multiple observers while the intra-observer agreement was calculated using Cohen’s kappa [16, 17]. The kappa values were interpreted using Table 1, as proposed in the literature [18]. For comparisons of intra- and inter-observer agreement between WLE and NBI, the rate of accordance between the first and second read of an image over the three observers and the standard error between the three observers were computed and then compared again between WLE and NBI with the Wilcoxon signed rank test.

**Table 1:** Interpretation of kappa values according to [18]

Kappa	Strength of agreement
< 0.00	Poor
0.00–0.20	Slight
0.21–0.40	Fair
0.41–0.60	Moderate
0.61–0.80	Substancial
0.81–1.00	Almost perfect

The mean sensitivity and specificity in WLE and NBI were further computed for subgroups of images with and without a previous laryngeal operation. These values were compared with the Mann–Whitney *U* test using the same rates per image and modality as described above. The distribution of malignant and benign diagnoses in both subgroups were compared with Pearson’s Chi-square test.

The statistical calculations used in this study were performed with IBM SPSS Statistics software package (version 24). The significance level was defined as *p* < 0.05.

## Results

After considering respective inclusion and exclusion criteria, 170 images with laryngeal lesions from 163 patients, were included in this study. The mean patient age at diagnosis was 56.5 years (Range 21–86 years). 75 images (44.1%) belonged to female and 95 (55.9%) to male patients.

According to the histopathology reports, 45 lesions (26.5%) were considered malignant (invasive carcinoma, carcinoma in situ and severe dysplasia) and 125 (73.5%) nonmalignant (all other diagnoses). The detailed distribution of the histopathological diagnoses and patient sex can be seen in Table 2.

**Table 2:** Distribution of histopathological diagnoses according to the status of previous laryngeal surgery (*n* = 170)

Diagnosis	Previous laryngeal surgery		Total
	Yes	No	
Invasive carcinoma	8	32	40 (23.5%)
Carcinoma in situ	2	0	2 (1.2%)
Severe dysplasia	2	1	3 (1.8%)
Moderate dysplasia	3	3	6 (3.5%)
Mild dysplasia	2	5	7 (4.1%)
Lipoma	0	1	1 (0.6%)
Papilloma	10	1	11 (6.5%)
Granuloma	0	6	6 (3.5%)
Hemangioma	0	3	3 (1.8%)
Polyp	0	18	18 (10.6%)
Cyst	0	16	16 (9.4%)
REINKE’s edema	0	28	28 (16.5%)
Hyper-/parakeratosis	2	13	15 (8.8%)
Chronic inflammation	3	11	14 (8.2%)
Total lesions	32 (18.9%)	138 (81.1%)	170 (100%)

The localization of the laryngeal lesion was the true vocal fold in most cases (151 lesions, 88.8%), followed by the false vocal fold (13 lesions, 7.6%), the arytenoid region (2 lesions, 1.2%), the laryngeal epiglottis (2 lesions, 1.2%), the aryepiglottic fold (1 lesion, 0.6%) and the subglottic region (1 lesion, 0.6%).

Previous laryngeal surgery was documented in 32 (18.9%) out of 170 lesions. The remaining 138 images (81.1%) belonged to patients with no documented previous laryngeal surgery. No further data about these operations was available. The distribution of histopathological diagnoses in these 32 images is presented in Table 2.

### Sensitivity, specificity, accuracy and DOR

An overview of the sensitivity, specificity, accuracy and DOR in diagnosing malignancy with flexible transnasal endoscopy in WLE and NBI can be seen in Table 3. The results show a moderate variation among the different observers. The sensitivity of NBI as an optical modality proved to be significantly higher (*p* < 0.05, 93.3%) than that of WLE (77.0%). The accuracy of NBI (96.3%) was also significantly higher (*p* = 0.001) than that of white light (92%). Both modalities had a specificity of 97.3%. The diagnostic odds ratio of NBI (501.83) was also higher than that of WLE (120.65).

**Table 3:** Sensitivity, specificity, accuracy and DOR for the diagnosis of malignancy with WLE and NBI

	White light endoscopy				Narrow band imaging				
	Obs.1	Obs.2	Obs.3	Mean (95%-CI)	Obs.1	Obs.2	Obs.3	Mean (95%-CI)	Significance level^a^
Sensitivity	84.4%	88.9%	57.8%	77.0% (68.8–85.3)	97.8%	97.8%	96.8%	93.3% (87.8–98.8)	p < 0.001
Specificity	96.8%	98.4%	84.4%	97.3% (95.6–99.1)	100%	99.2%	92.8%	97.3% (95.6–99.1)	p = 1.000
Accuracy	93.5%	95.9%	86.5%	92.0% (89.1–94.8)	99.4%	98.8%	90.5%	96.3% (94.3–98.2)	p = 0.001
DOR	164.2	492.0	41.4	120.7	2600.0	5456.0	70.0	501.8	n.a

*DOR* diagnostic odds ratio, *n.a*. not applicable, *CI* confidence interval

^a^The significance level refers to the mean values

### Inter- and intra-observer agreement

The inter-observer agreement as depicted in the Fleiss’ kappa value was substantial (kappa = 0.661) for WLE and almost perfect (kappa = 0.849) for NBI. The agreement was significantly higher in the evaluation with NBI compared to WLE (*p* = 0.008).

The kappa values for the intra-observer agreement for WLE and NBI are presented in Table 4. The intra-observer agreement in WLE was almost perfect for two of the observers and substantial for the third one and almost perfect for all three observers in NBI. The kappa values in the evaluation with NBI were higher over all three observers compared to WLE, though not significantly so (*p* = 0.531).

**Table 4:** Intraobserver agreement for the diagnosis of malignancy with WLE and NBI

	WLE		NBI	
	Cohen’s kappa	Standard error	Cohen’s kappa	Standard error
Observer 1	0.924	0.074	1.000	0.000
Observer 2	0.844	0.105	1.000	0.000
Observer 3	0.739	0.141	0.864	0.093

Significance level: *p* = 0.531 for WLE vs. NBI over all three observers

### Previous laryngeal surgery and differences in sensitivity/specificity

The sensitivity and specificity of the subgroups divided based on the history of previous laryngeal surgery are presented in Table 5. In the subgroup with no history of laryngeal surgery, the sensitivity was significantly higher both in WLE (*p* = 0.042) and NBI (*p* = 0.006) compared to the sensitivity in the subgroup with previous surgery. The specificity showed no significant differences.

**Table 5:** Comparison of sensitivity and specificity of subgroups based on the history of previous laryngeal surgery

	With previous laryngeal surgery (*n* = 32) (%)	Without previous laryngeal surgery (*n* = 138) (%)	Significance level
White light endoscopy			
Sensitivity	63.9	81.8	*p* = 0.042
Specificity	95.0	97.8	*p* = 0.149
Narrow band imaging			
Sensitivity	86.1	96.0	*p* = 0.006
Specificity	96.7	97.5	*p* = 0.609

Comparing WLE with NBI, in the subgroup without a history of laryngeal surgery the sensitivity was significantly higher in NBI (*p* < 0.001). Meanwhile in the subgroup with previous surgery, the sensitivity was higher in NBI, trending towards significance (*p* = 0.094). The specificities showed no significant differences (*p* = 1.000).

The proportion of malignant lesions was slightly larger in the subgroup with previous surgery (37.5% compared to 23.9% in the subgroup without, but this difference was not statistically significant (*p* = 0.125 in Pearson’s Chi-square test).

## Discussion

Laryngeal cancer belongs to the most common head and neck cancer entities with a prognosis dependent on the clinical stage at the time of diagnosis [19]. More than 90% are histologically squamous cell carcinomas [20]. The prognosis remains poor for more advanced stages, and so early diagnosis was a greater focus in the last years [6, 21]. Computed axial tomography (CAT) and magnetic resonance imaging (MRI) are valuable tools in the diagnostic staging of laryngeal cancer, but they nevertheless mostly fail to detect early stage cancer (Tis, T1 < 10 mm) or laryngeal preneoplastic lesions. Laryngeal white light endoscopy (WLE), with flexible or rigid endoscopes, followed by microlaryngoscopy (MLS) for histopathologic assessment is widely accepted as the standard diagnostic modality for benign and malignant laryngeal lesions [20, 22]. Despite technological developments in the field, endoscopic diagnosis of preneoplastic lesions and early carcinoma is still challenging, such as when vocal fold leukoplakia is present [23–25].

NBI is a horizontal image-enhancement technique which can provide examiners with more information about the epithelial and vascular changes of laryngeal lesions and surrounding mucosa [2]. The evaluation of vascular changes in in the context of early diagnosis of cancer of a profound role, since the formation of new vessels (neoangiogenesis) is known to be an important hallmark in tumor development [26, 27]. As shown before, the morphology of these vascular alterations can be indicative of the histology of the respective lesion [13, 28–31]. To use the clinical information provided by NBI optimally in the context of laryngology, the evaluation of a triplet, consisting of epithelial changes, vascular changes, and a possible volume change of the vocal fold has recently been proposed [14].

Several publications in various clinical settings proliferate the use of NBI in laryngology. It has been reported that NBI can ease the endoscopic diagnosis of laryngeal papillomatosis and other benign lesions [32–34]. There is also evidence that the addition of NBI to WLE can lead to better identification of laryngeal cancer [12, 28] and its recurrences [35]. Assessing vocal fold leukoplakia, the NBI vessel pattern has been strongly correlated to the final histology [25, 36]. Similarly, other studies show a diagnostic benefit for NBI in the differentiation between laryngeal cancer and other lesions, but do not exclusively include laryngeal lesions [37]. In the context of screening, De Vito et al. designed a prospective study for the diagnosis of laryngeal carcinoma in a risk group of patients with dysphonia and/or reflux symptoms and long-term exposure to tobacco and/or alcohol. Using NBI, malignant and premalignant laryngeal lesions were diagnosed with a sensitivity of 97% and a specificity of 92.5% [38]. Nevertheless, as most studies use different inclusion and exclusion criteria, only partial comparisons are possible [9] and there is a dearth of evidence from multiple-observer studies [8, 39].

Our study design allows for the inclusion of a wide range of benign, malignant and dysplastic lesions combined with a multiple-observer assessment of images with known histological diagnosis. The sensitivity of NBI was 93.3% in our data; i.e. similar to the pooled sensitivity of 94% published in the meta-analysis of Sun et al., while the specificity of 97.3% versus a pooled specificity of 89% was much higher in our data [7]. The increase in sensitivity in NBI compared to WLE, with no statistically significant differences in specificity, has also been described by other authors [12, 28]. On the contrary, Shoffel-Havakuk et al. reported a decrease of specificity with NBI (61.19% versus 76,1%) [40]. A low specificity has been attributed in some cases to over-diagnosis with NBI because there is a lack of experience with the modality [9, 12]. This could not be observed in our results.

Concerning observer reliability, Zwakenberg et al. found an increase of intra- and inter-observer agreement in less- and more-experienced observers when combining NBI with WLE [8]. The images were taken intraoperatively and classified from the observers in two categories: benign or malignant. Nogués‑Sabaté et al. presented similar results for images obtained with a flexible endoscope, while the observers classified the lesions in three categories: benign, dysplastic or malignant [39]. In both publications, the study population included lesions other than laryngeal, such as oropharyngeal and hypopharyngeal lesions. The parameters of sensitivity, specificity or accuracy of each modality were also not assessed [8, 39].

According to our data, the inter-observer agreement increased from substantial (kappa = 0.661) in WLE to almost perfect (kappa = 0.849) in NBI. Although these data indicate high observer reliability for both WLE and NBI, it should be noted that the agreement between observers is not to be confused with the diagnostic value of each modality. Nevertheless, NBI leads to an increase in the agreement of clinical judgements between observers, as shown in previous studies in laryngology [8, 39]. The intra-observer agreement was almost perfect for two of our observers, both in WLE and NBI. For the third observer, the intra-observer agreement was substantial for WLE and almost perfect for NBI. Possible reasons for the higher kappa values in comparison to previous publications could be that our study population was more homogenous, exclusively consisting of laryngeal lesions and the involvement of only experienced observers. The variance of histological diagnoses in different studies could also influence the levels of agreement. Contrary to previous publications, a different strategy was used in our study concerning the calculation of intra-observer agreement. As due to time considerations only a portion of the images was presented for a second read, comparability to other studies is limited.

Previous laryngeal surgery could negatively affect the endoscopic diagnosis of laryngeal dysplasia and cancer, as they can lead to structural changes in the laryngeal tissue and to the development of scars [22, 41]. The loss of elastic fibers and the development of scar tissue can lead to limitations in the accuracy of the clinical assessment, as previously reported for the autofluorescence endoscopy [22]. This has a profound clinical relevance, for example in the posttreatment follow-up and the detection of recurrence. On the other hand, NBI has been reported to be superior to autofluorescence in that respect [42]. We believe this is the first study to compare the sensitivity and specificity of diagnosis in relation to the history of previous laryngeal surgery. Our data showed a significant difference in the sensitivity (63.9% vs. 81.9% for WLE, 86.1% vs. 97.0% for NBI) but no significant differences in specificity, strengthening the hypothesis that previous laryngeal surgery can make the endoscopic diagnosis of laryngeal malignancy more difficult. Comparing the distribution of diagnoses in both subgroups, we found that the proportion of malignant lesions was slightly larger in the subgroup with previous surgery (37.5% compared to 23.9% in the subgroup without), but this difference was not statistically significant. Furthermore, we observed a higher sensitivity of NBI compared to WLE in both subgroups. As these results are partly based on small subgroups of images, further investigation should be performed to validate them.

The combination of data on the diagnostic value of the endoscopic assessment (sensitivity, specificity, accuracy, diagnostic odds ratio) with data on the reliability of the assessment (inter- and intra-observer agreement) is the main strength of this study. Furthermore, the inclusion of benign, premalignant and malignant lesions provided a realistic context close to the everyday practice of otorhinolaryngologists. In this way, a selection bias could be eliminated and we could calculate the specificity of both modalities. WLE and NBI images could be independently evaluated, in contrast to a concurrent use of WLE, which is a common bias in other studies [9]. Limitations of this study include the use of still images for the evaluation of laryngeal lesions, and retrospective design.

In everyday practice, assessment of morphology of laryngeal lesions must be complemented with a functional examination of the vocal folds e.g. using videostroboscopy or high-speed glottography. NBI belongs to the group of horizontal endoscopic techniques, allowing a superficial evaluation of lesions, making it ideal for screening. Other horizontal techniques include autofluorescence, hyperspectral imaging, SPIES and i-SCAN [9, 43]. Additionally, vertical diagnostic modalities such as optical coherence tomography and endoscopic sonography supply valuable information about the vertical extension of the lesion. For specific questions, cellular techniques such as contact endoscopy can allow in vivo and in situ study of cellular characteristics [44]. In that context, combinations of different modalities have been reported as promising and could be introduced in clinical routine in the future [43, 45, 46].

The results of this study are consistent with literature and demonstrate the benefits of flexible transnasal endoscopy in the diagnosis of benign, premalignant and malignant laryngeal lesions, allowing the recommendation for widespread use in everyday practice. Additionally, narrow band imaging demonstrated superiority to white light endoscopy in the detection of malignancy in terms of sensitivity, accuracy and DOR, while maintaining a high specificity. The high levels of intra- and inter-observer agreement exhibit the validity of the above data. Flexible transnasal endoscopy, with or without NBI is, therefore, a valuable tool with many clinical applications such as in screening, laryngeal examination of dysphonia, differential diagnosis, and oncologic post treatment follow-up. Although the histological examination remains the gold standard for the evaluation of laryngeal lesions, an adequate endoscopic examination can guide patient selection and reduce unnecessary biopsies.
